# Deep Coupled Integration of CSAC and GNSS for Robust PNT

**DOI:** 10.3390/s150923050

**Published:** 2015-09-11

**Authors:** Lin Ma, Zheng You, Bin Li, Bin Zhou, Runqi Han

**Affiliations:** 1 Department of Precision Instrument, Tsinghua University, Beijing 100084, China; E-Mails: yz-dpi@mail.tsinghua.edu.cn (Z.Y.); libin@mail.tsinghua.edu.cn (B.L.); zhoubin98@tsinghua.org.cn (B.Z.); hanrunqi91@163.com (R.H.); 2 State Key Laboratory of Precision Measurement Technology and Instruments, Tsinghua University, Beijing 100084, China

**Keywords:** integration, CSAC, GNSS, weighted linear optimal estimation, Kalman filter

## Abstract

Global navigation satellite systems (GNSS) are the most widely used positioning, navigation, and timing (PNT) technology. However, a GNSS cannot provide effective PNT services in physical blocks, such as in a natural canyon, canyon city, underground, underwater, and indoors. With the development of micro-electromechanical system (MEMS) technology, the chip scale atomic clock (CSAC) gradually matures, and performance is constantly improved. A deep coupled integration of CSAC and GNSS is explored in this thesis to enhance PNT robustness. “Clock coasting” of CSAC provides time synchronized with GNSS and optimizes navigation equations. However, errors of clock coasting increase over time and can be corrected by GNSS time, which is stable but noisy. In this paper, weighted linear optimal estimation algorithm is used for CSAC-aided GNSS, while Kalman filter is used for GNSS-corrected CSAC. Simulations of the model are conducted, and field tests are carried out. Dilution of precision can be improved by integration. Integration is more accurate than traditional GNSS. When only three satellites are visible, the integration still works, whereas the traditional method fails. The deep coupled integration of CSAC and GNSS can improve the accuracy, reliability, and availability of PNT.

## 1. Introduction

Positioning, navigation, and timing (PNT) technology involves technologies that concern time and space. In modern society, people rely more on PNT than on other technologies in any historical period. In fact, the questions “when and where” are one of the most central problems that involve PNT. Global navigation satellite systems (GNSSs) are the most widely used PNT technology. GNSS satellites construct a navigation platform and provide high precision position, velocity, and time information for various types of military or civilian users. Although the PNT service provided by GNSS has the advantage of all-weather performance and no error accumulation, radio signals, which are transmitted from GNSS satellites to the ground, are weak because of long transmission distances and limited transmission power [1,2,3,4]. As such, GNSS cannot provide effective PNT services in physical blocks, such as in a natural canyon, canyon city, underground, underwater, and indoors. In such situations, the visible satellite number does not meet the requirement of having a value of more than 4. Thus, the GNSS receiver is unable to employ its navigation function.

An atomic clock can provide high-precision atomic frequency standard, which is the highest accuracy that is humanly possible [5]. The atomic clock uses quantum transition energy of atoms or molecules. The transition frequency is the reference standard on which the local oscillator is locked [6]. The frequency of electromagnetic waves emitted or absorbed by the atomic transition is stable, which is beneficial to the quantization character of atomic transition energy. With the development of micro-electromechanical system (MEMS) technology, the chip scale atomic clock (CSAC) gradually matures, and performance is constantly improved [7,8,9,10,11,12]. Therefore, the size, weight, power, and cost (SWaP + C) of CSAC has considerably improved. Typically, the second stability of CSAC can reach 10^−10^. In 2002, the USA National Institute of Standard and Technology developed a physical component of the CPT atomic clock fabricated by using MEMS technology. Its volume was only 1 cm^3^. This CSAC has a power consumption of 120 mW, a physical size of 40.6 mm × 35.5 mm × 11.4 mm, better than 1.5 × 10^−10^@ 1 s stability, and better than 5 × 10^−11^@ 10 s stability [13]. CSAC was successfully commercialized and provides great convenience for the extensive application of the atomic frequency standard. In the PNT field, the CSAC atomic frequency standard can replace the traditional crystal. This feature not only improves the accuracy of timing but also provides significant advantages for positioning.

Generally, a low-cost oscillator is used in a GNSS receiver. The clock offset between receiver and satellite can be determined by navigation equations, while the clock can be synchronized with the satellite. Based on this fact, the receiver can use a low-cost oscillator for PNT. If a more stable clock is used in the receiver, then clock offset can be predicted. Consequently, the navigation equation of the unknown will be reduced to 3. Three satellites can provide PNT. If the value of time of arrival (TOA) is still more than 4, then redundancy TOA can improve the accuracy, reliability, and availability of PNT. In the early stage of GNSS, few satellites remain in orbit. As such, the researchers focused on clock-aided GNSS and made some achievements. Sturz [14] derived vertical dilution of precision (VDOP) and horizontal dilution of precision (HDOP) of three satellites and an atomic clock. The stability of atomic clocks enabled the atomic clock to expand the availability of three satellites for PNT. Van Graas [15] proved that adding an atomic clock was more valuable than adding a satellite for three-satellite navigation. The effect of the added atomic clock is significant, especially for the application of high vertical position accuracy, such as aircraft landing systems. Misra [16] proposed a clock model and proved that using an atomic clock can significantly reduce VDOP and slightly reduce HDOP because of high relevance between vertical position error and receiver clock offset. Kline [17] improved the clock model using precision carrier phase measurement and calculated VDOP variation of atomic clock-aided GNSS. The vertical position accuracy of atomic clock-aided GNSS and usual situations was compared by performing a fly test. Zhang [18] used adaptive low-pass filter to estimate rubidium atomic clock offset and showed that atomic clock-aided GNSS works efficiently in single point or differential situations. Bednarz [19] considered that clock offset has the same effect on all pseudo ranges and results in vertical position change.

The literature focuses on traditional rubidium clock-aided GNSS. A traditional rubidium clock has the advantage of high stability, which is beneficial to the integrated system. However, it is costly, large, heavy, and energy consuming. By contrast, CSAC is cheap, small, light, and energy efficient, but is unstable. Therefore, the interaction between CSAC and GNSS needs to be researched. This thesis explores deep coupled integration of CSAC and GNSS to enhance PNT robustness. The second section discusses the theoretical aspect of a coupled relationship. The third section implements some simulations. The fourth section carries out fixed point tests, and the last section provides some conclusions.

## 2. Deep Coupled Integration in Theory

**Figure 1:**
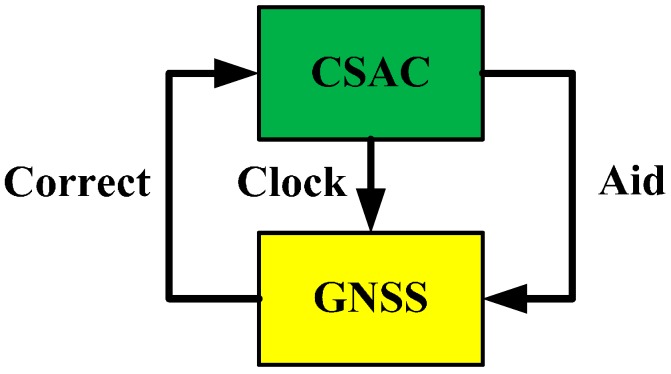
Coupled relationship between Chip Scale Atomic Clock (CSAC) and Global Navigation Satellite Systems (GNSS).

Typically, the clock offset can be determined if GNSS constellation geometry is good. In most cases, the clock offset can be a few nanoseconds. As such, the initial state of time integral can be determined. In addition, this initial state can be determined by eternal equipment, such as GNSS timing unit or ground timing service center. “Clock coasting” of CSAC provides time synchronized with GNSS time and optimizes navigation equations. However, errors of clock coasting increase over time and can be corrected by GNSS time, which is stable but noisy. Figure 1 describes the coupled relationship between CSAC and GNSS. This section discusses their integration in theory. This section is divided into three parts. In the first part, traditional GNSS positioning principles are listed, and some important concepts are introduced. In the second part, weighted linear optimal estimation is used to construct navigation equations of CSAC-aided GNSS. In the third part, CSAC correction by GNSS is discussed.

### 2.1. GNSS Receiver Positioning Principles

After the GNSS receiver tracks the radio signals transmitted by satellites, it obtains navigation messages, calculates the satellite positions, linearizes the observation equations, solves pseudo ranges, and uses least square or Kalman filter algorithm to determine the receiver position.

Pseudo range observation equations are as follows:

(1) $$\rho^{\left(n\right)}=r^{\left(n\right)}+\delta t_{u}-\delta t^{\left(n\right)}+I^{\left(n\right)}+T^{\left(n\right)}+Rot^{\left(n\right)}+M^{\left(n\right)}+\varepsilon_{\rho }^{\left(n\right)}$$

where n=1,2,⋯N, n is the visible satellite identifier, r is the geometric distance between the satellite and the receiver, $\delta t_{u}$ is the receiver clock offset that is equivalent to length, δt is the satellite clock offset, I is the ionosphere delay, T is the troposphere delay, $Rot^{\left(n\right)}$ is the earth rotation correction, and $M^{\left(n\right)}$ is the sum of the ephemeris errors, phase center variation error, and multipath error. ε is the pseudo range measurement noise.

Offset corrected pseudo range measurement $\rho_{c}^{\left(n\right)}$ is

(2) $$\rho_{c}^{\left(n\right)}=\rho^{\left(n\right)}+\delta t^{\left(n\right)}-I^{\left(n\right)}-T^{\left(n\right)}-Rot^{\left(n\right)}-M^{\left(n\right)}$$

Without consideration of noise $\varepsilon_{\rho }^{\left(n\right)}$, navigation equations are established as follows:

(3) $$\left\{ \begin{array}{l} \sqrt{{\left(x^{\left(1\right)}-x\right)}^{2}+{\left(y^{\left(1\right)}-y\right)}^{2}+{\left(z^{\left(1\right)}-z\right)}^{2}}+\delta t_{u}=\rho_{c}^{\left(1\right)}\\ \sqrt{{\left(x^{\left(2\right)}-x\right)}^{2}+{\left(y^{\left(2\right)}-y\right)}^{2}+{\left(z^{\left(2\right)}-z\right)}^{2}}+\delta t_{u}=\rho_{c}^{\left(2\right)}\\ \cdots \cdots \cdots \\ \sqrt{{\left(x^{\left(N\right)}-x\right)}^{2}+{\left(y^{\left(N\right)}-y\right)}^{2}+{\left(z^{\left(N\right)}-z\right)}^{2}}+\delta t_{u}=\rho_{c}^{\left(N\right)} \end{array}\right.$$

The initial state $\left[x_{0};\delta t_{u,0}\right]$ and $x_{0}=\left[\begin{array}{ccc} x_{0} & y_{0} & z_{0} \end{array}\right]$ are set. The matrix equation at $\left[x_{k-1};\delta t_{u,k-1}\right]$ is linearized when k is the step counter of Newton iterations. Least square algorithm is used to determine unknown parameters

(4) $$\left[\begin{array}{c} \Delta x\\ \Delta \delta t_{u} \end{array}\right]=\left[\begin{array}{c} \Delta x\\ \Delta y\\ \Delta z\\ \Delta \delta t_{u} \end{array}\right]={\left(G^{\text{T}}G\right)}^{-1}G^{\text{T}}b$$

where

(5) $$G=\left[\begin{array}{cccc} -1_{x}^{\left(1\right)}(x_{k-1}) & -1_{y}^{\left(1\right)}(x_{k-1}) & -1_{z}^{\left(1\right)}(x_{k-1}) & 1\\ -1_{x}^{\left(2\right)}(x_{k-1}) & -1_{y}^{\left(2\right)}(x_{k-1}) & -1_{z}^{\left(2\right)}(x_{k-1}) & 1\\ \vdots & \vdots & \vdots & \vdots \\ -1_{x}^{\left(N\right)}(x_{k-1}) & -1_{y}^{\left(N\right)}(x_{k-1}) & -1_{z}^{\left(N\right)}(x_{k-1}) & 1 \end{array}\right]=\left[\begin{array}{cc} M & O \end{array}\right]$$

(6) $$b=\left[\begin{array}{c} \rho_{c}^{\left(1\right)}-r^{\left(1\right)}(x_{k-1})-\delta t_{u,k-1}\\ \rho_{c}^{\left(2\right)}-r^{\left(2\right)}(x_{k-1})-\delta t_{u,k-1}\\ \vdots \\ \rho_{c}^{\left(N\right)}-r^{\left(N\right)}(x_{k-1})-\delta t_{u,k-1} \end{array}\right]$$

In Equation (5), $\left[\begin{array}{ccc} -1_{x}^{\left(n\right)}(x_{k-1}) & -1_{y}^{\left(n\right)}(x_{k-1}) & -1_{z}^{\left(n\right)}(x_{k-1}) \end{array}\right]$ is the line of sight between the visible satellite and the receiver. $O\text{=}{\left[\begin{array}{cccc} 1 & 1 & \cdots & 1 \end{array}\right]}_{1\times N}^{\text{T}}$.

The subscript URE is the user range error. The covariance matrix of the positioning error can be written as follows:

(7) $$\text{Cov}\left(\begin{array}{c} \varepsilon_{x}\\ \varepsilon_{y}\\ \varepsilon_{z}\\ \varepsilon_{\delta t_{u}} \end{array}\right)=\text{E}\left(\left[\begin{array}{c} \varepsilon_{x}\\ \varepsilon_{y}\\ \varepsilon_{z}\\ \varepsilon_{\delta t_{u}} \end{array}\right]\left[\begin{array}{cccc} \varepsilon_{x} & \varepsilon_{y} & \varepsilon_{z} & \varepsilon_{\delta t_{u}} \end{array}\right]\right)={\left(G^{\text{T}}G\right)}^{-1}\sigma_{\text{URE}}^{2}=H\sigma_{\text{URE}}^{2}$$

Dilution of precision (DOP) describes the magnified relationship between measurement error and positioning error. DOP can be obtained from coefficient matrix H.

Geometric dilution of precision (GDOP), position dilution of precision (PDOP), HDOP, VDOP, and time dilution of precision (TDOP) are given as follows:

(8) $$\text{GDOP=}\sqrt{h_{11}+h_{22}+h_{33}+h_{44}}$$

(9) $$\text{PDOP=}\sqrt{h_{11}+h_{22}+h_{33}}$$

(10) $$\text{HDOP=}\sqrt{h_{11}+h_{22}}$$

(11) $$\text{VDOP=}\sqrt{h_{33}}$$

(12) $$\text{TDOP=}\sqrt{h_{44}}$$

where $h_{ii}$(i=1,2,3,4) is the diagonal elements of the matrix H.

Equation (5) can be partitioned as follows:

(13) $$H={\left(G^{\text{T}}G\right)}^{-1}={\left[\begin{array}{cc} M^{\text{T}}M & M^{\text{T}}O\\ MO^{\text{T}} & N \end{array}\right]}^{-1}=\left[\begin{array}{cc} H_{\text{PDOP}} & \\ & H_{\text{TDOP}} \end{array}\right]$$

According to inverse of block matrix theorem, which is shown in the Appendix [20], and a comparison between Equation (13) and the inverse form of the block matrix, Equations (13) and (14) can be derived. The subscript letter “H” indicates that the DOPs are derived from H.

(14) $${\text{PDOP}}_{H}={\left(M^{\text{T}}M\right)}^{-1}+\frac{{\left(M^{\text{T}}M\right)}^{-1}M^{\text{T}}OO^{\text{T}}M{\left(M^{\text{T}}M\right)}^{-1}}{N-O^{\text{T}}M{\left(M^{\text{T}}M\right)}^{-1}M^{\text{T}}O}$$

(15) $${\text{TDOP}}_{H}=\frac{1}{N-O^{\text{T}}M{\left(M^{\text{T}}M\right)}^{-1}M^{\text{T}}O}$$

Xie [21] provides detailed derivation processes of GNSS receiver positioning principles and explains those equations above.

### 2.2. Performance of CSAC

Stability is an important parameter of a clock and can be described by Allan variance [22].

(16) $$\sigma^{2}(\tau )=\langle \frac{{\left[\phi (t+2\tau )-2\phi (t+\tau )+\phi (t)\right]}^{2}}{2\tau \omega_{0}}\rangle$$

where $\sigma^{2}(\tau )$ is the Allan variance, τ is the averaging time (s), ϕ(t) is the clock signal phase at time *t* (rad), $\omega_{0}$ is the natural frequency of the clock (rad/s), and 〈〉 is an infinite time average operator.

Define

(17) $${\bar{y}}_{k}=\frac{\phi (t_{k}+\tau )-\phi (t_{k})}{2\tau \omega_{0}}$$

Allan variance can be written as

(18) $$\sigma^{2}(\tau )=\frac{1}{2(N-1)}\sum_{k=1}^{N-1}{\left({\bar{y}}_{k+1}-{\bar{y}}_{k}\right)}^{2}$$

Where N is the sample number. Allan deviation σ(τ) is typically used to express clock stability.

Table 1 shows comparisons of different kinds of clocks. Compared with traditional atomic clocks, CSAC has advantages of power consumption, size, and cost. Compared with a crystal oscillator, CSAC has the advantage of stability.

**Table 1:** Comparisons of different kinds of clocks.

	Stability τ = 10 s	Power Consumption	Size	Cost
PXO	10^−5^~10^−6^	<10 mW	<50 mm^3^	<USD 1
TXCO	10^−6^~10^−7^	~15 mW	~50 mm^3^	~USD 10
OXCO	10^−7^~10^−8^	~2.5 W	~20 cm^3^	~USD 200
**CSAC**	**10^−10^**~**10^−11^**	~**90 mW**	~**17 cm^3^**	~**USD 1500**
Rubidium atomic clock	10^−11^	~10 W	~200 cm^3^	~USD 2000

### 2.3. Weighted Linear Optimal Estimation of CSAC-Aided GNSS

CSAC clock coasting provides accuracy time information in a short period of time. It can be used in navigation equations and change the traditional method of positioning.

If the receiver clock offset is known, then Equation (1) can be written as follows:

(19) $${\overset{⌢}{\rho }}_{c}^{\left(n\right)}=[\rho^{\left(n\right)}+\delta t^{\left(n\right)}-I^{\left(n\right)}-T^{\left(n\right)}-Rot^{\left(n\right)}-M^{\left(n\right)}]-[\delta t_{u}]$$

The pseudo range error can be divided into two parts. One part is the error described by Equation (2), and the other is the pseudo range error caused by the receiver clock noise.

(20) $$\Delta {\overset{⌢}{\rho }}_{c}^{\left(n\right)}=\Delta \rho_{c}^{\left(n\right)}+\varepsilon_{\delta t_{u}}$$

Equation (20) describes the covariance of the pseudo range error.

(21) $$\mathrm{cov}(\Delta {\overset{⌢}{\rho }}_{c})=\mathrm{cov}(\Delta \rho_{c}+\Delta \delta t_{u}\cdot O)=\sigma_{\text{URE}}^{2}I+\sigma_{clock}^{2}OO^{\text{T}}$$

where $\sigma_{clock}^{2}$ is the receiver clock noise variance, and $\sigma_{\text{URE}}^{2}$ is the pseudo range error covariance, which does not include the receiver clock error.

If we define $c\equiv \frac{\sigma_{clock}^{2}}{\sigma_{\text{URE}}^{2}}$, Equation (21) can be written as follows:

(22) $$\mathrm{cov}(\Delta {\overset{⌢}{\rho }}_{c})=\sigma_{\text{URE}}^{2}(I+cOO^{\text{T}})$$

Weighted objective functions are designed as follows:

(23) $$R(\Delta x)={\left(\Delta {\overset{⌢}{\rho }}_{c}-M\Delta x\right)}^{\text{T}}\cdot w\cdot (\Delta {\overset{⌢}{\rho }}_{c}-M\Delta x)$$

where weighted function w is a N×N matrix. To keep R a positive definite matrix, w should be a Hermitian matrix.

The objective function is minimized by imposing

(24) $$\frac{\partial R(\Delta x)}{\partial \Delta x}{|}_{\Delta x=\Delta {\overset{⌢}{x}}_{wls}}=-2M^{\text{T}}\cdot w\cdot {\left(\Delta {\overset{⌢}{\rho }}_{c}-M\Delta x\right)}^{\text{T}}{|}_{\Delta x=\Delta {\overset{⌢}{x}}_{wls}}=0$$

This gets

(25) $$\Delta {\overset{⌢}{x}}_{wls}={\left(M^{\text{T}}wM\right)}^{-1}M^{\text{T}}w\Delta {\overset{⌢}{\rho }}_{c}$$

Error equations of weighted linear optimal estimation are seen in Equation (25). Optimal *w* should be selected to get the minimum $Cov[\Delta {\overset{⌢}{x}}_{wls}]$.

(26) $$\begin{array}{l} Cov[\Delta {\overset{⌢}{x}}_{wls}]=E[(\Delta x-\Delta {\overset{⌢}{x}}_{wls}){\left(\Delta x-\Delta {\overset{⌢}{x}}_{wls}\right)}^{\text{T}}]\;\\ \;=E\left\{[(\Delta x-{\left(M^{\text{T}}wM\right)}^{-1}M^{\text{T}}w\Delta \overset{⌢}{\rho }][{\left(\Delta x-{\left(M^{\text{T}}wM\right)}^{-1}M^{\text{T}}w\Delta \overset{⌢}{\rho }\right]}^{\text{T}}\right\}\\ \;=E\{\left[(\Delta x-{\left(M^{\text{T}}wM\right)}^{-1}M^{\text{T}}w(M\Delta x+n)\right]\\ \;*[{\left(\Delta x-{\left(M^{\text{T}}wM\right)}^{-1}M^{\text{T}}w(M\Delta x+n)\right]}^{\text{T}}\}\\ \;=E\left\{[-{\left(M^{\text{T}}wM\right)}^{-1}M^{\text{T}}wn][-{\left(M^{\text{T}}wM\right)}^{-1}M^{\text{T}}wn){]}^{\text{T}}\right\}\\ \;={\left(M^{\text{T}}wM\right)}^{-1}M^{\text{T}}w(E[nn^{\text{T}}])wM{\left(M^{\text{T}}wM\right)}^{-1}\\ \;={\left(M^{\text{T}}wM\right)}^{-1}M^{\text{T}}wCov(\Delta \overset{⌢}{\rho })wM{\left(M^{\text{T}}wM\right)}^{-1} \end{array}$$

With the use of the matrix Cauchy-Schwarz inequality theorem shown in the Appendix, Equation (26) can be rewritten as follows:

(27) $$\begin{array}{l} Cov[\Delta {\overset{⌢}{x}}_{wls}]={\left(M^{\text{T}}wM\right)}^{-1}M^{\text{T}}wV_{n}wM{\left(M^{\text{T}}wM\right)}^{-1}=B^{\text{T}}B\\ \;\geq {\left(AB\right)}^{\text{T}}{\left(AA^{\text{T}}\right)}^{-1}(AB)={\left(AA^{\text{T}}\right)}^{-1}={\left(M^{\text{T}}V_{n}^{-1}M\right)}^{-1} \end{array}$$

If *w_opt_* = $V_{n}^{-1}$, then Inequality Equation (27) becomes an equality, and $Cov[\Delta {\overset{⌢}{x}}_{wls}]$ has its minimum value.

Then

(28) $$Cov[\Delta {\overset{⌢}{x}}_{wls}]={\left(M^{\text{T}}V_{n}^{-1}M\right)}^{-1}$$

(29) $$\Delta {\overset{⌢}{x}}_{wls}={\left(M^{\text{T}}V_{n}^{-1}M\right)}^{-1}M^{\text{T}}V_{n}^{-1}\Delta \overset{⌢}{\rho }$$

Therefore, the optimal weighted coefficient is *w_opt_* = $V_{n}^{-1}$.

Equations (22) and (28) give

(30) $$Cov[\Delta {\overset{⌢}{x}}_{wls}]=\sigma_{\text{URE}}^{2}{\left[M^{\text{T}}{\left(I+cOO^{\text{T}}\right)}^{-1}M\right]}^{-1}$$

where

(31) $${\left(I+cOO^{\text{T}}\right)}^{-1}=I-\frac{cOO^{\text{T}}}{1+cO^{\text{T}}O}=I-\frac{cOO^{\text{T}}}{1+cN}$$

In this case

(32) $$Cov[\Delta {\overset{⌢}{x}}_{wls}]=\sigma_{\text{URE}}^{2}{\left(M^{\text{T}}M-\frac{cM^{\text{T}}OO^{\text{T}}M}{1+cN}\right)}^{-1}$$

Define now

(33) $$\begin{array}{l} {\text{PDOP}}_{W}\equiv {\left(M^{\text{T}}M-\frac{cM^{\text{T}}OO^{\text{T}}M}{1+cN}\right)}^{-1}\\ \;={\left(M^{\text{T}}M\right)}^{-1}+\frac{c{\left(M^{\text{T}}M\right)}^{-1}M^{\text{T}}OO^{\text{T}}M{\left(M^{\text{T}}M\right)}^{-1}}{1+cN-cO^{\text{T}}M{\left(M^{\text{T}}M\right)}^{-1}M^{\text{T}}O} \end{array}$$

${\text{PDOP}}_{W}$ describes the PDOP of CSAC-aided GNSS based on weighted linear optimal estimation algorithm.

(34) $$\begin{array}{l} \Delta \text{PDOP}\equiv {\text{PDOP}}_{H}-{\text{PDOP}}_{W}\\ \;=\frac{{\left(M^{\text{T}}M\right)}^{-1}M^{\text{T}}OO^{\text{T}}M{\left(M^{\text{T}}M\right)}^{-1}}{N-O^{\text{T}}M{\left(M^{\text{T}}M\right)}^{-1}M^{\text{T}}O}-\frac{c{\left(M^{\text{T}}M\right)}^{-1}M^{\text{T}}OO^{\text{T}}M{\left(M^{\text{T}}M\right)}^{-1}}{1+cN-cO^{\text{T}}M{\left(M^{\text{T}}M\right)}^{-1}M^{\text{T}}O}\\ \;=\frac{{\left(M^{\text{T}}M\right)}^{-1}M^{\text{T}}OO^{\text{T}}M{\left(M^{\text{T}}M\right)}^{-1}}{\left(1+cN-cO^{\text{T}}M{\left(M^{\text{T}}M\right)}^{-1}M^{\text{T}}O)(N-O^{\text{T}}M{\left(M^{\text{T}}M\right)}^{-1}M^{\text{T}}O\right)}\\ \;=\frac{{\left(M^{\text{T}}M\right)}^{-1}M^{\text{T}}OO^{\text{T}}M{\left(M^{\text{T}}M\right)}^{-1}}{\left(1+cN-cO^{\text{T}}M{\left(M^{\text{T}}M\right)}^{-1}M^{\text{T}}O\right)}{\text{TDOP}}_{H} \end{array}$$

If CSAC is an ideal clock, then $\sigma_{clock}^{2}=0$ and *c* = 0. As such, Equation (34) becomes Equation (35)

(35) $$\begin{array}{l} \Delta \text{PDOP}=\frac{{\left(M^{\text{T}}M\right)}^{-1}M^{\text{T}}OO^{\text{T}}M{\left(M^{\text{T}}M\right)}^{-1}}{N-O^{\text{T}}M{\left(M^{\text{T}}M\right)}^{-1}M^{\text{T}}O}\\ \;={\left(M^{\text{T}}M\right)}^{-1}M^{\text{T}}OO^{\text{T}}M{\left(M^{\text{T}}M\right)}^{-1}\times {\text{TDOP}}_{H} \end{array}$$

If CSAC is faulty, then $\sigma_{clock}^{2}\to \infty$ and c→∞, ΔPDOP=0.

### 2.4. GNSS-Corrected CSAC

After the receiver obtains its position information, the electromagnetic wave propagation delay can be determined, and one pulse per second (1PPS) can be recovered. The stability of clocks on GNSS satellites is better than 10^−14^. Ground monitoring stations use master atomic clock signals, which has greater precision than satellite clocks in calibrating the clocks on satellites. Therefore, 1PPS, which is based on the satellite clock, has good long-term stability. However, 1PPS is affected by electromagnetic wave transmission error and receiver thermal noise. The short-term stability of 1PPS is not good. By contrast, CSAC has good short-term stability but mediocre long-term stability compared with 1PPS. Good long-term and short-term stability can be achieved when CSAC and 1PPS signals are combined.

Kalman filter is used to combine complementary characteristics. The theory model of CSAC is the state equation of Kalman filter, and 1PPS constructs the observation equation. This arrangement has the benefit of enabling the prediction of CSAC error when 1PPS is lost.

The phase offset of CSAC can be described as follows:

(36) $$p(t)=p_{0}+p_{1}(t-t_{0})+\frac{1}{2}p_{2}{\left(t-t_{0}\right)}^{2}+\xi_{1}(t)+\xi_{2}(t)$$

where p is the phase offset, $p_{0}$ is the initial phase offset, $p_{1}$ is the frequency offset, $p_{2}$ is the frequency drift, $\xi_{1}$ is the phase noise, and ξ_2_ is the measurement noise.

CSAC state equation is then constructed. Let

(37) $$X(k)=\left[\begin{array}{ccc} x_{1}(k) & x_{2}(k) & x_{3}(k) \end{array}\right]=\left[\begin{array}{ccc} p_{0}(k) & p_{1}(k) & p_{2}(k) \end{array}\right]$$

(38) [x1(k+1)x2(k+1)x3(k+1)]=[1c12c201c001]·[x1(k)x2(k)x3(k)]+[Δx1Δx2Δx3]

where $\Delta x_{1}$ is the phase noise, $\Delta x_{2}$ is the frequency noise, and $\Delta x_{3}$ frequency drift noise. The variances are $q_{1}=\sigma_{1}^{2}$, $q_{2}=\sigma_{2}^{2}$, $q_{3}=\sigma_{3}^{2}$. c is the state transition time constant, and *c* = 1.

The observation equation can be written as follows:

(39) $$Z(k)=x_{1}(k)+n_{0}(k)$$

where $n_{0}$ is the measurement noise and its variance is $q_{4}=\sigma_{4}^{2}$.

In existing literature, the noise covariance matrix is given by Equation (39) [23]. The variances of $q_{1}$, $q_{2}$, $q_{3}$ can be determined by Equation (40) [24,25]. *h*_2_, *h*_0_, and $h_{-2}$ are slope coefficients of the Allan variance plot of CSAC. $f_{h}$ is the high-frequency cutoff, which is defined as the upper limit in the spectral bandwidth of CSAC.

(40) $$\begin{array}{l} P=E\left[\left[\begin{array}{c} \Delta x_{1}\\ \Delta x_{2}\\ \Delta x_{3} \end{array}\right]\cdot \left[\begin{array}{ccc} \Delta x_{1} & \Delta x_{2} & \Delta x_{3} \end{array}\right]\right]\\ \;=\left[\begin{array}{ccc} q_{1}c+q_{2}c^{3}/3+q_{3}c^{5}/20 & q_{2}c^{2}/2+q_{3}c^{4}/8 & q_{3}c^{3}/6\\ q_{2}c^{2}/2+q_{3}c^{4}/8 & q_{2}c+q_{3}c^{3}/3 & q_{3}c^{2}/2\\ q_{3}c^{3}/6 & q_{3}c^{2}/2 & q_{3}c \end{array}\right] \end{array}$$

(41) $$\begin{array}{l} q_{1}=\frac{f_{h}}{{\left(2\pi \right)}^{2}}\cdot h_{2}\\ q_{2}=\frac{1}{2}\cdot h_{0}\\ q_{3}=\pi^{2}\cdot h_{-2} \end{array}$$

## 3. Simulations

**Figure 2:**
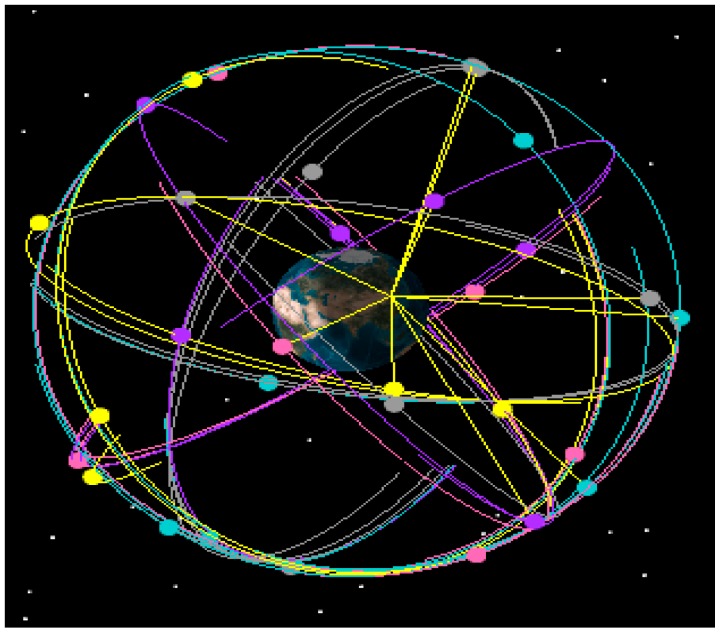
GNSS orbit model.

**Figure 3:**
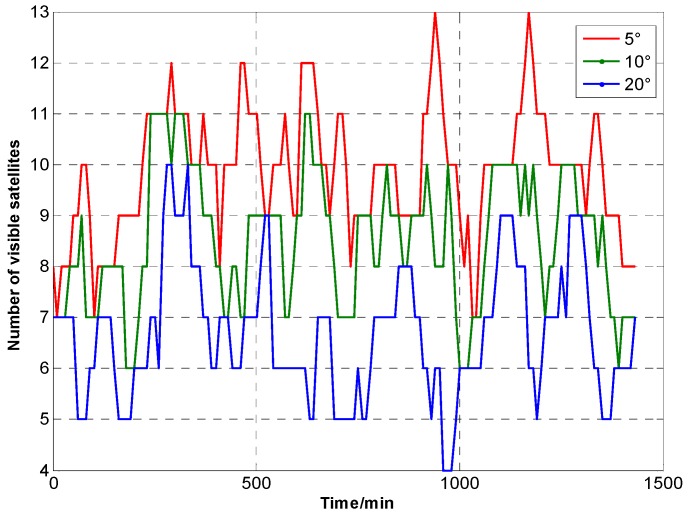
Visibility of GNSS satellites at different cutoff angles.

**Figure 4:**
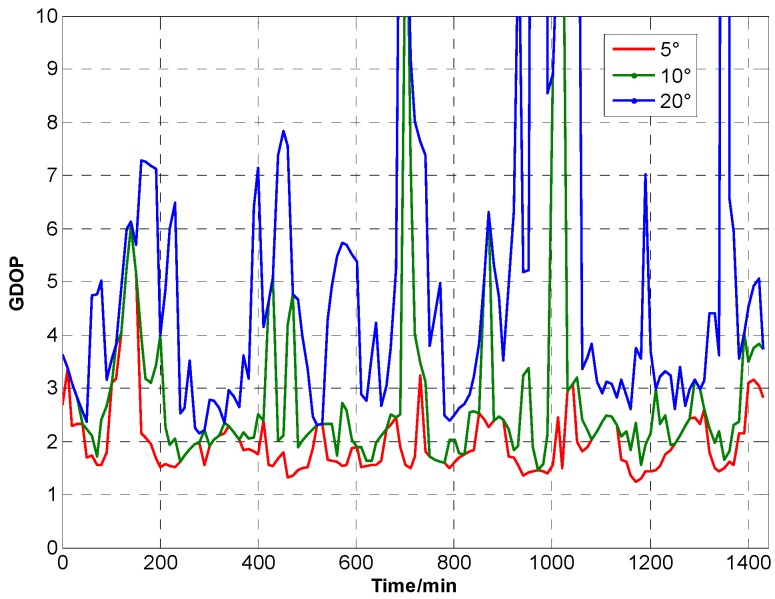
GDOP at different cutoff angles.

A satellite simulation tool is used to construct the GNSS satellite orbit model. GPS is used as GNSS constellation. The simulations is based Beijing, China, which is located at 39.9062° N, 116.388° E, as an example. Simulation time is from 12:00:00, 1 January 2015 to 12:00:00, 2 January 2015. The time interval is 10 min. Figure 2 shows the GNSS satellite model. Figure 3 and Table 2 show GNSS visibility and average DOP. Figure 4 shows GDOP at different cutoff angles. Typically, VDOP is 1.5 to 2 times greater of HDOP. As the cutoff angle increases, average visible number sharply decreases. By contrast, DOPs quickly increase, which implies that the position accuracy is poor. Taking 5° as reference, DOPs at 10° are 1.5 times larger, whereas DOPs at 20° are roughly five times.

**Table 2:** Average visible number of satellite and DOPs at different cutoff angles.

Cutoff Angle	Average Visible Number	GDOP	PDOP	HDOP	VDOP	TDOP
5°	9.82	2.02	1.79	0.98	1.49	0.93
10°	8.53	2.90	2.50	1.28	2.13	1.46
20°	6.66	10.23	8.21	3.91	7.18	6.03

For the ideal CSAC, *c* = 0. DOPs of the integrated and traditional GNSS are compared at different cutoff angles. Figure 5, Figure 6, Figure 7, Figure 8, Figure 9, Figure 10, Figure 11, Figure 12 and Figure 13 show the simulation results. Table 3 lists their comparisons.

**Figure 5:**
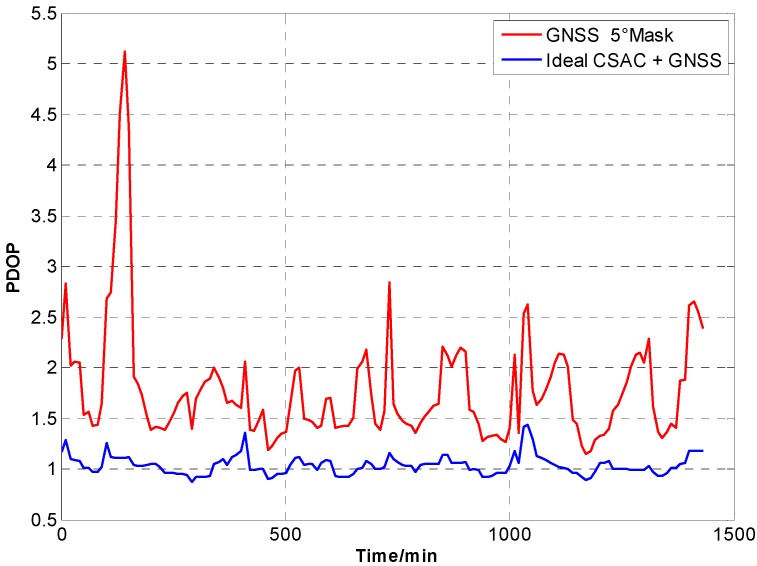
PDOP comparison at 5° cutoff.

**Figure 6:**
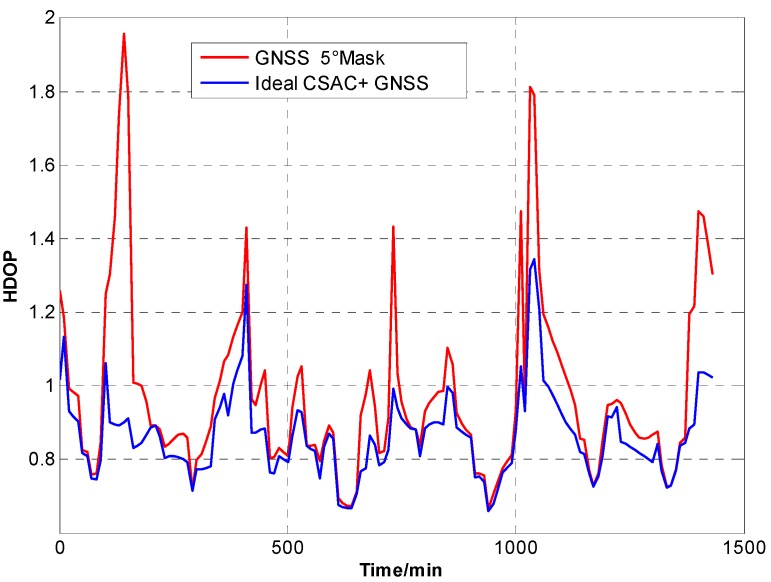
HDOP comparison at 5° cutoff.

**Figure 7:**
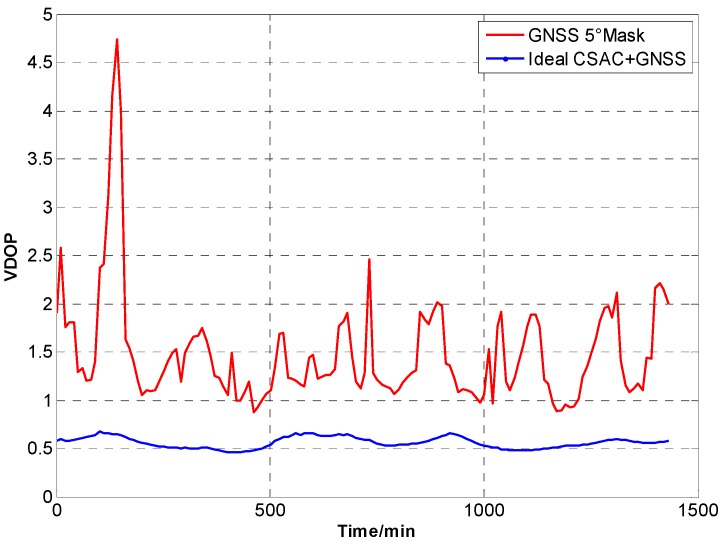
VDOP comparison at 5° cutoff.

**Figure 8:**
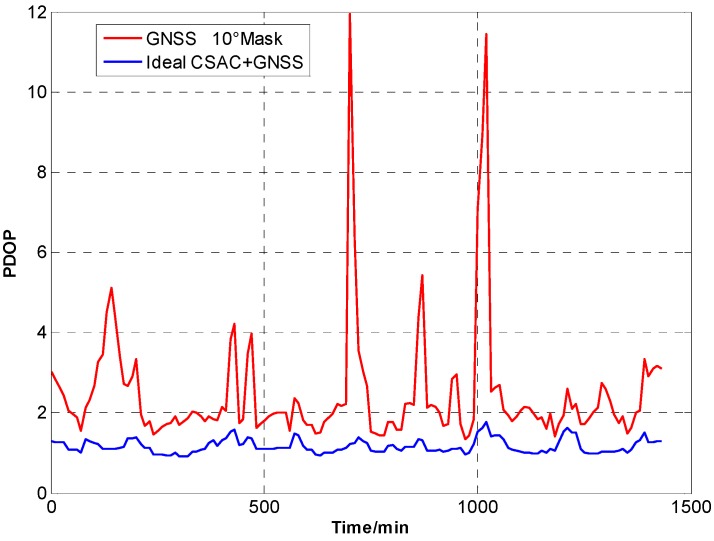
PDOP comparison at 10° cutoff.

**Figure 9:**
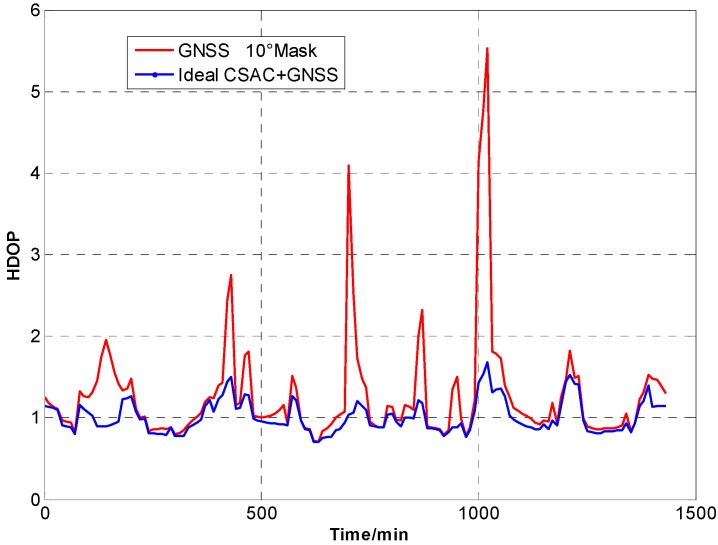
HDOP comparison at 10° cutoff.

**Figure 10:**
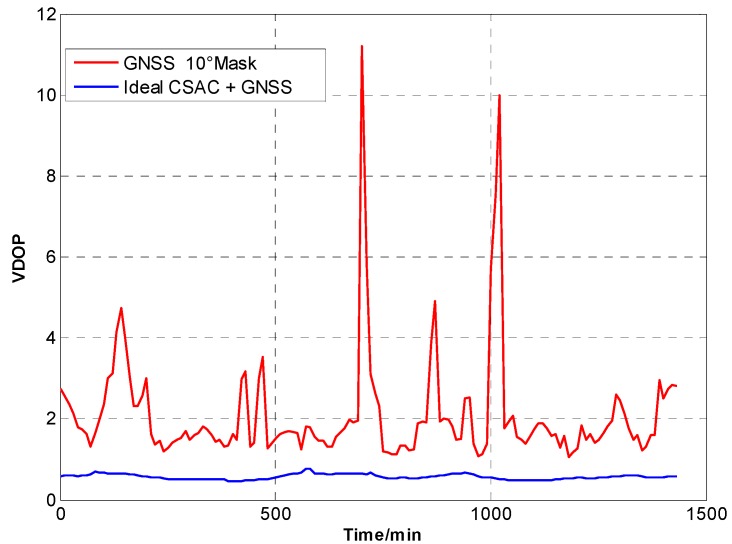
VDOP comparison at 10° cutoff.

**Figure 11:**
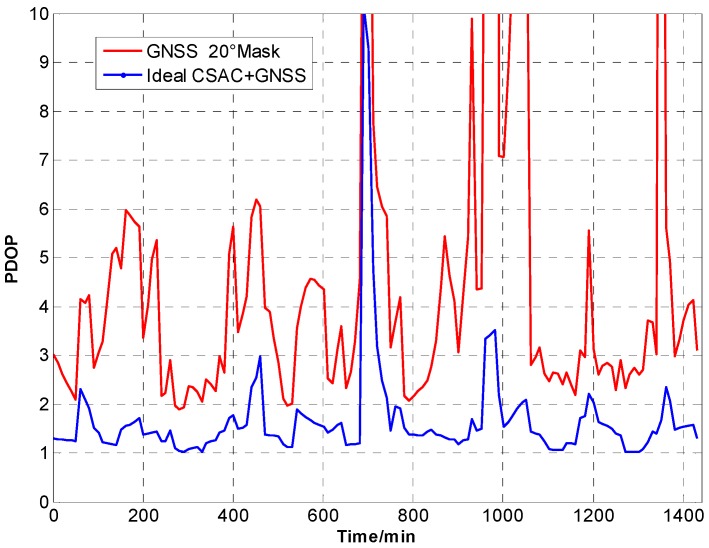
PDOP comparison at 20° cutoff.

**Figure 12:**
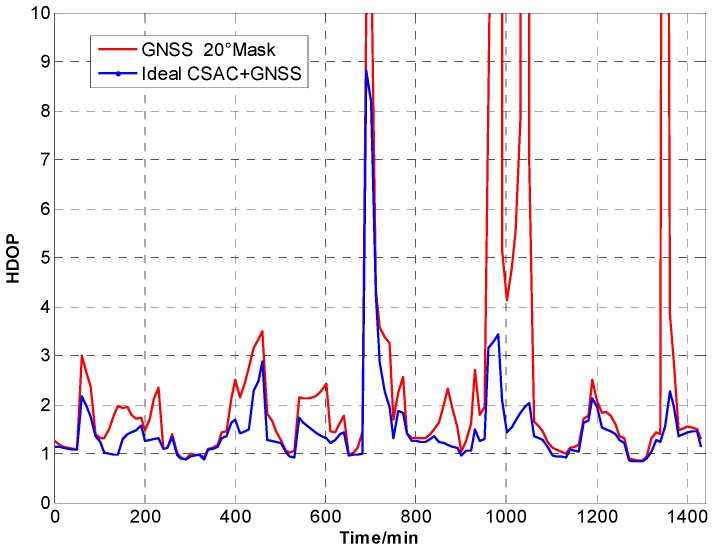
HDOP comparison at 20° cutoff.

**Figure 13:**
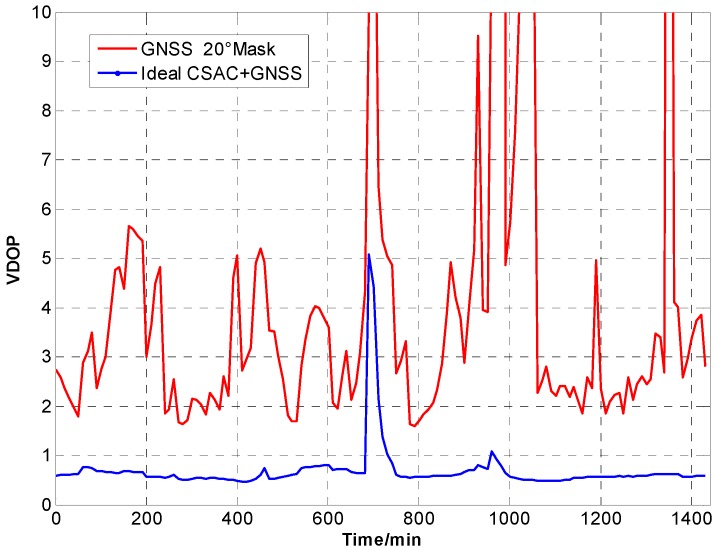
VDOP comparison at 20° cutoff.

**Table 3:** DOP comparison between Ideal CSAC + GNSS and traditional GNSS.

Cutoff Angle	Average Visible Number	ΔPDOP		ΔHDOP		ΔVDOP	
		Average	Max	Average	Max	Average	Max
5°	9.82	0.75	4.02	0.11	1.06	0.93	4.09
10°	8.53	1.34	12.97	0.27	4.95	1.56	12.70
20°	6.66	6.54	2850	2.39	1309	6.50	2532

A number of conclusions can be drawn from the simulation results. CSAC is beneficial to all DOPs and significantly decreases VDOP compared with HDOP. As the cutoff angle increases, a visible number of satellites decrease and DOP worsens. CSAC can also significantly improve DOPs at a large cutoff angle significantly; this result indicates that CSAC is effective at worse DOPs and improves positioning accuracy considerably. Taking 5° as reference, average ΔDOPs at 10° are two times larger, whereas average ΔDOPs at 20° are roughly 10 times larger. From the point of maximum value, ΔDOPs at 20° can reach thousands.

**Figure 14:**
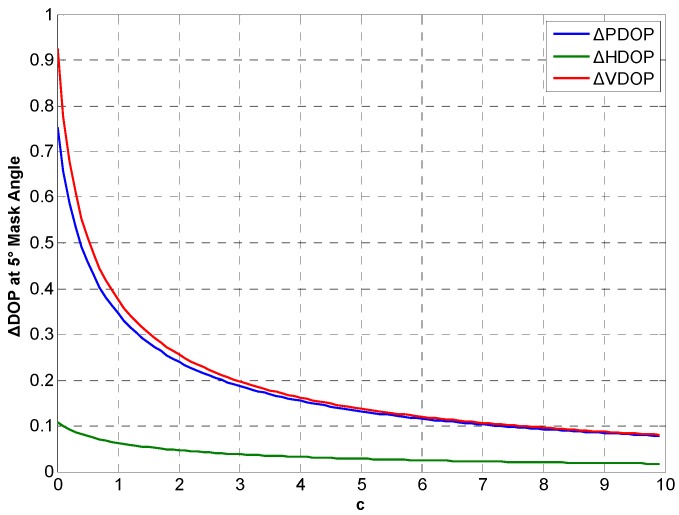
ΔDOP with different *c* at 5° cutoff angle.

**Figure 15:**
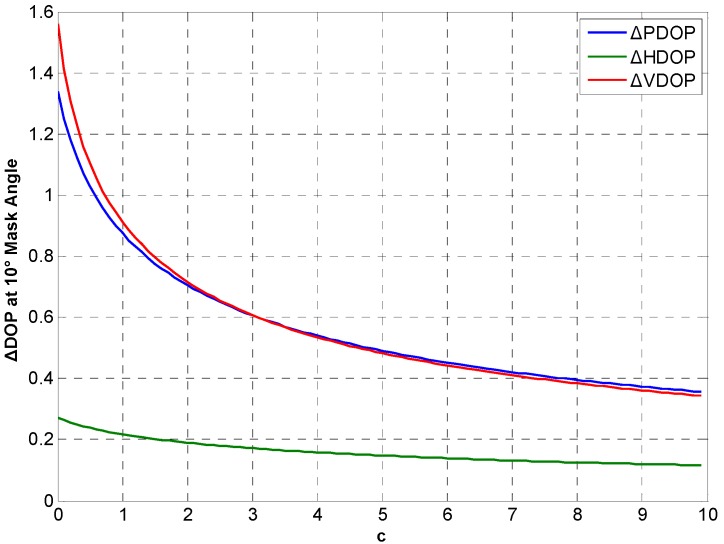
ΔDOP with different *c* at 10° cutoff angle.

**Figure 16:**
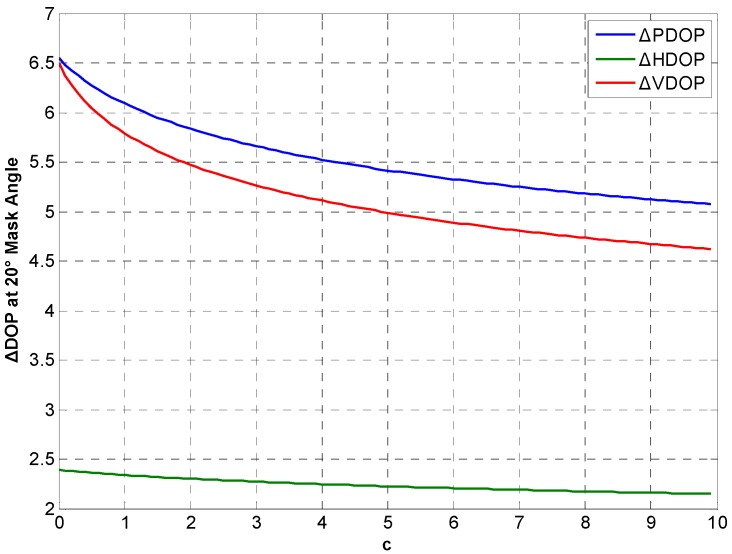
ΔDOP with different *c* at 20° cutoff angle.

Considering real CSAC, c>0 and c is determined by noise intensity. DOP variations between the integration and traditional at different *c* in Beijing for 24 h are simulated. Figure 14, Figure 15 and Figure 16 show ΔDOP with different *c* at different degree cutoff angles. The following conclusions can be drawn from the simulation results: As the noise intensity increases, DOP improvement of the integration gradually decreases. In addition, DOP improvement shows the same downward trend at different cutoff angles.

## 4. Experimental Section

**Figure 17:**
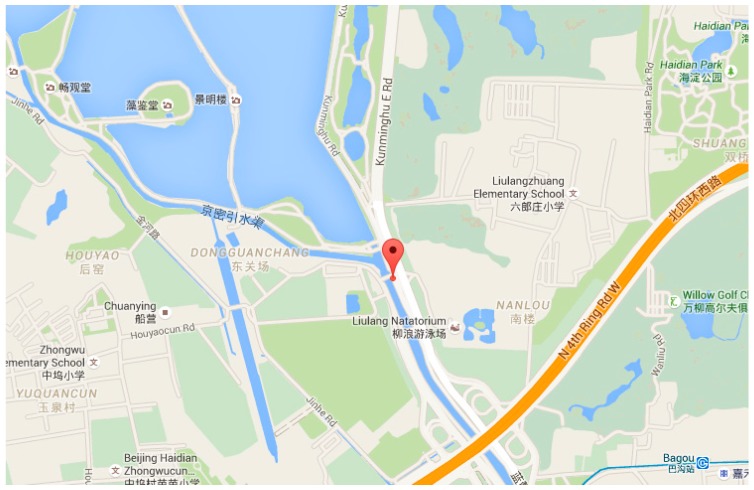
Experiment site in the map.

**Figure 18:**
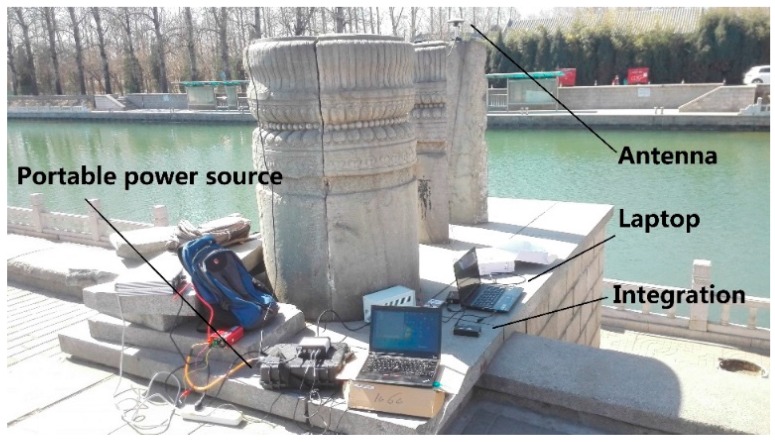
Experiment scene.

Fixed single position experiments are designed to verify the integration of CSAC and GNSS. The experiments were carried out at Kunming Lakeside in Summer Palace, Beijing, China. The mark in Figure 17 is the experiment site; Figure 18 illustrates the scene.

**Figure 19:**
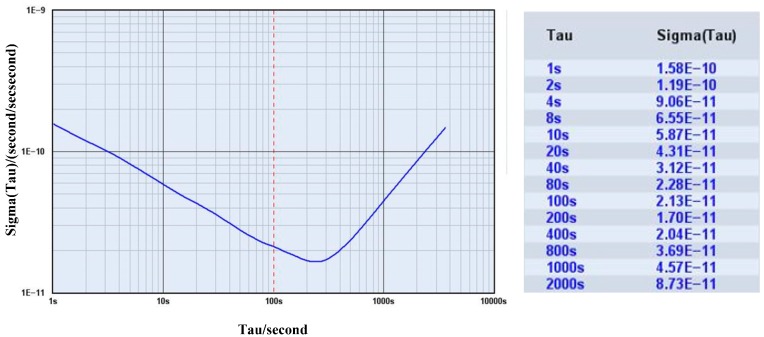
Allan root deviation of CSAC measured by Symmetricom 3120A.

SA.45 CSAC, which is manufactured by Symmetricom, is used for the experiment. The performance of CSAC is measured by using a high-performance Phase Noise Test Probe Symmetricom 3120A. Figure 19 shows the results from 3120A. The typical stability of CSAC is 1.58 × 10^−10^ at 1 s, 5.87 × 10^−11^ at 10 s, and 2.13 × 10^−11^ at 100 s as given by Allan root deviation.

Paolo [26] lists some mass-market receivers such as LEA EVK-5T from U-Blox and NVS NV08-CSM from Leica Geosystems. However, in order to deeply integrate the CSAC and GNSS, GNSS intermediate frequency collector and software defined receiver are used, which are more flexible than mass-market receivers. The type of chip for GNSS intermediate frequency collection is MAX2769, and USB 3.0 technology is used to transmit data to a laptop. The antenna is HX-CS5601A, which is manufactured by Shenzhen Harxon Antenna Technology Co., Ltd. in Shenzhen, China. The output frequency of CSAC is 10 MHz. A GNSS intermediate frequency collector with a crystal oscillator is at 16.369 MHz. The laptop saves the data of the collector. The data processing software is a software-defined receiver created by the laboratory in Tsinghua University.

Table 4 shows comparisons of the integration and the GNSS collector, which uses a crystal oscillator. The site position information is obtained by Google Maps. The integration has good accuracy and precision positioning, especially with the height direction.

**Table 4:** Comparisons of the integration and GNSS with crystal oscillator.

	Latitude		Longitude		Height	
	Mean/°	Std/m	Mean/°	Std/m	Mean/m	Std/m
Site position	39.979092	-	116.274708	-	54	-
Integration	39.979084	3.49	116.274713	4.66	53	7.69
GNSS	39.979125	5.30	116.274696	6.65	43	12.96

The matrix G in Equation (5) can be determined as

(42) $$G=\left[\begin{array}{cccc} 0.217823 & -0.927923 & -0.288886 & 1\\ 0.789554 & -0.552636 & -0.251531 & 1\\ 0.742365 & 0.019585 & -0.663967 & 1\\ -0.369786 & -0.919573 & -0.100745 & 1\\ 0.583853 & -0.777678 & 0.216286 & 1\\ -0.014635 & -0.509891 & -0.855466 & 1\\ -0.830094 & -0.349515 & -0.426517 & 1\\ -0.647133 & -0.170355 & -0.738310 & 1 \end{array}\right]$$

**Figure 20:**
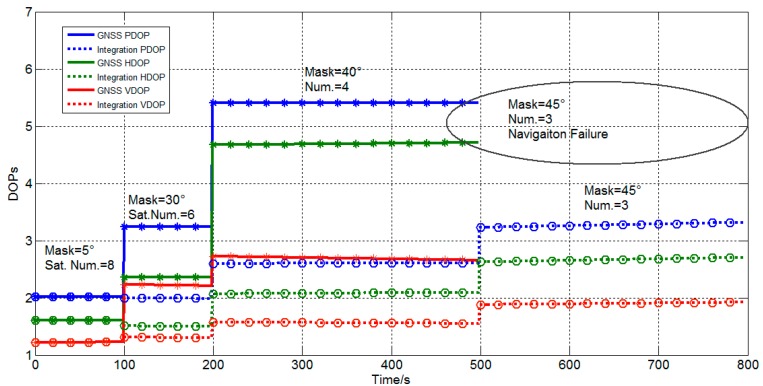
DOPs of integration and GNSS.

The cutoff angle is set by the software to simulate the problem of shade from other objects. As shown in Figure 20 and Table 5, from 0–100 s, the cutoff angle is 5°, and the visual satellite number is 8. From 100–200 s, the cutoff angle is 30°, and the visual satellite number is 6. From 200–500 s, the cutoff angle is 40°, and the visual satellite number is 4. From 500–800 s, the cutoff angle is 45°, and the visual satellite number is 3. When the visual satellite number is less than 4, traditional GNSS which uses least square method fails, whereas the integration still works well. The DOPs of GNSS and integration are plotted. The standard deviations are listed in Table 5. The comparisons indicate that DOPs of integration are smaller than GNSS and that the accuracy of the positioning improved.

**Table 5:** Comparison of Integration and GNSS.

		0 s–100 s	100 s–200 s	200 s–500 s	500 s–800 s
Cutoff/°		5	30	40	45
Visible Sat. Num.		8	6	4	3
Integration	Std E	3.49	4.54	8.51	9.33
	Std N	4.66	6.08	10.09	13.07
	Std U	7.69	8.27	11.96	14.85
GNSS	Std E	5.30	7.49	13.80	Inf
	Std N	6.65	10.13	121.42	Inf
	Std U	12.96	28.36	133.47	Inf

## 5. Conclusions

CSAC and GNSS integration is discussed in this paper. The weighted linear optimal estimation algorithm is used in CSAC-aided GNSS, while Kalman filter is used in GNSS-corrected CSAC. Simulations show that the integration can improve DOPs, especially for VDOP. For high DOP situations, the integration can significantly decrease DOPs. As the noise intensity increases, the improvement gradually decreases. Fixed position tests are carried out. Results show that the integration is more accurate than the traditional method. When only three satellites are visible, the integration still works well, whereas the traditional method fails. Therefore, the deep coupled integration of CSAC and GNSS can enhance PNT robustness.

## Appendix A

### A.1. Inverse of Block Matrix Theorem

Assume that $P=\left[\begin{array}{cc} A & B\\ C & D \end{array}\right]$, A is n order matrix, B and C are n×m and m×n matrixes, respectively, and D is m order matrix.

If A is invertible, then P is invertible $\Leftrightarrow D-CA^{-1}B$ is invertible.

$$P^{-1}=\left[\begin{array}{cc} A^{-1}+A^{-1}B{\left(D-CA^{-1}B\right)}^{-1}CA^{-1} & -A^{-1}B{\left(D-CA^{-1}B\right)}^{-1}\\ -{\left(D-CA^{-1}B\right)}^{-1}CA^{-1} & {\left(D-CA^{-1}B\right)}^{-1} \end{array}\right]$$

### A.2. Matrix Cauchy-Schwarz Inequality Theorem

Assume A and B are m×n and n×l matrixes, respectively. Then, $AA^{\text{T}}$ is invertible.

$$B^{\text{T}}B\geq {\left(AB\right)}^{\text{T}}{\left(AA^{\text{T}}\right)}^{-1}(AB)$$

If and only if a m×l matrix P and $B=A^{\text{T}}P$ exists, then the inequality becomes an equality.
